# A study on prevalence of anemia and growth pattern among HIV infected children and adolescents from rural areas attending VCTC and ART center Govt. General Hospital, Vijayawada, A.P., India

**DOI:** 10.1186/1471-2334-14-S3-P55

**Published:** 2014-05-27

**Authors:** Baratha Jyothi Naladi, Sunita Kanikaram

**Affiliations:** 1 Department of Zoology and Aquaculture, Acharya Nagarjuna University, Nagarjunanagar, Guntur, Andhra Pradesh, India

## Background

Globally, the HIV epidemic remains a serious challenge, and continues to take its toll particularly on vulnerable populations such as children and adolescents. However, background co-morbidities compound the problem in affected populations in India. Two such major co-morbidities include anemia and poor nutrition.

## Methods

The present article deals with the profile of HIV infected children and adolescents in HAART era and Pre-HAART era who were attending the ART centre, Govt. General Hospital, Vijayawada.

## Results

Totally 125 subjects of age group 1-20 years were included in the study. Among 125 HIV+ subjects only 45 subjects were in HAARTera, 75 subjects in pre-HAART era and 5 subjects died during the study period. The study patients in HAART era showed a significant increase (*p*<0.001) in CD4 counts from 174 to 902 cells/cmm, significant decrease (*p*<0.001) in Hb content from 7.3 to 6.5 gm/dL and significant increase (*p*<0.05) in BMI level from 18.1 to 22.6 from baseline to follow up treatment after 18 months. The study patients in pre-HAART era showed a significant increase (*p*<0.05) in mean CD4 counts from 592 to 790 cells/cmm from initial count to count after 18 months. The subjects in pre-HAART era showed low level of Hb content 6.9 gm/dL in male and 6.3 gm/dL in female subjects; the mean BMI level was 20.43 in male and 18.47 in female subjects.

## Conclusion

Our study reinforces the finding that anemia, growth failure and malnutrition are major manifestations of HIV infection in Indian children with prognostic significance.

